# Unusual Mycobacterium marinum Infection in a Heart Transplant Recipient: A Case Report

**DOI:** 10.7759/cureus.32387

**Published:** 2022-12-10

**Authors:** Julia M Nelson, Purvi Patel, Maryjka Blaszczyk, Deepa Iyer, Pinki Bhatt, Ahmed Abdul Azim

**Affiliations:** 1 Medicine, Rutgers Robert Wood Johnson Medical School, New Brunswick, USA; 2 Infectious Diseases, Allergy, and Immunology, Rutgers Robert Wood Johnson Medical School, New Brunswick, USA; 3 Pathology and Laboratory Medicine, Rutgers Robert Wood Johnson Medical School, New Brunswick, USA; 4 Cardiovascular Medicine, Rutgers Robert Wood Johnson Medical School, New Brunswick, USA

**Keywords:** nontuberculous mycobacteria, marinum, reactivation, latency, heart transplant, mycobacterium marinum

## Abstract

*Mycobacterium marinum *(*M. marinum*)* *is a species of nontuberculous mycobacteria that is a rare cause of disease in humans and is usually associated with aquatic exposures. Symptoms manifest, on average, three weeks after exposure, although cases with longer incubation periods have been reported in the literature. Herein, we describe an unusual case presentation of an *M. marinum *infection in the left upper extremity of a heart transplant recipient. The case is notable for its prolonged incubation period and for being the first documented case of *M. marinum *infection in a heart transplant recipient. We hypothesize that, given the patient's immunosuppressive medication regimen in the post-transplant period, this case could represent a reactivation phenomenon of a latent infection.

## Introduction

*Mycobacterium marinum* (*M. marinum*) is a species of nontuberculous mycobacteria that was first identified as a fish pathogen but later discovered to cause human disease [1,2]. The disease most commonly manifests as a cutaneous papulonodular lesion on the upper extremity; however, it can also track lymphatically and develop a sporotrichoid appearance [3-5]. Other documented manifestations of the disease include osteomyelitis, tenosynovitis, septic arthritis, and rarely, in immunocompromised individuals, disseminated infection [6-9]. The incidence of *M. marinum* infection has been estimated between 0.04 and 0.27 per 100,000 per year, with the vast majority of cases (>80%) associated with exposures to aquatic environments or marine life [4, 10-12]. Disease symptoms usually manifest within a few weeks of exposure [11]. Herein, we describe an unusual case presentation of a *M. marinum* skin and soft tissue infection, notable for its prolonged incubation period as well as for being the first documented case of *M. marinum* in a heart transplant recipient.

## Case presentation

A 68-year-old male with a recent history of orthotopic heart transplant (OHT) for non-ischemic cardiomyopathy presented to the emergency department with left upper extremity skin lesions after being referred by his transplant cardiologist. Approximately six weeks prior to presentation, the patient noticed several small vesicles on the left forearm, which progressively enlarged over a period of weeks to become nodular, indurated, non-tender, non-pruritic, and productive of serosanguinous drainage. Over that period, the lesions began to track from the medial wrist to the epitrochlear region, and he developed areas of subcutaneous induration in the forearm. The patient denies any additional symptoms associated with the lesions. He had no prior history of or exposure to others with similar lesions. He exercised caution after the transplant and denied any recent exposure to animals, fish tanks, plants, or new soaps or detergents. Of note, the patient is a retired marine biologist and prior to his OHT, an avid fisher; however, he states that his last fishing trip or potential marine exposure was approximately two months prior to his OHT and six months prior to this current presentation.

**Figure 1:**
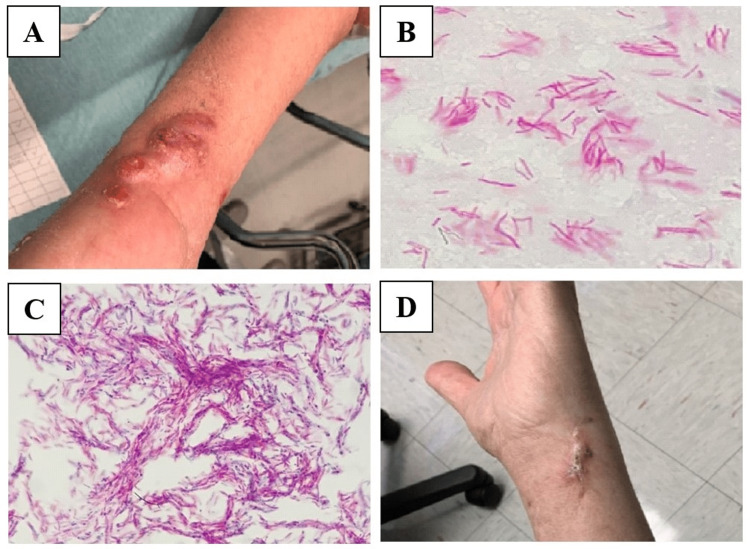
Mycobacterium marinum skin lesions, histological sections, and cultures A. Papulonodular lesions of the left upper extremity early in the clinical presentation; B. Histological section of skin biopsy stained with acid-fast bacteria (AFB) stain showing numerous AFB-positive mycobacteria, original magnification x 1000; C. Culture of skin lesions stained with Kinyoun stain showing dense AFB-positive mycobacteria forming cords, original magnification x 1000; D. Picture of left upper extremity following six months of antimicrobial therapy

A physical exam revealed several raised, indurated skin nodules ranging from two to four centimeters in diameter along the medial aspect of the left distal forearm, a similar lesion at the lateral epitrochlear region, and two crusted lesions on the dorsal forearm (Figure 1A). Notably, there was a large area of non-tender skin induration over the medial forearm, which was "woody" in character. Other than mild hypertension, the remainder of the physical exam was unremarkable.

**Figure 2:**
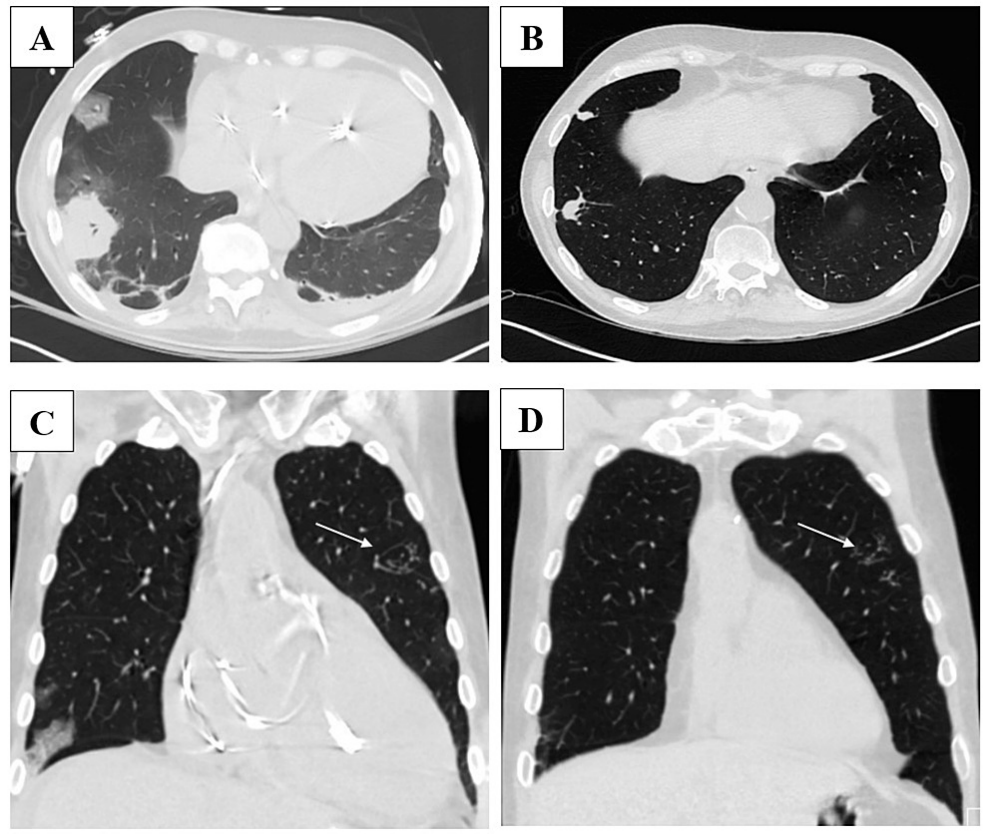
Chest CT findings demonstrating improvement in the right middle and lower lobe necrotic lung lesions and persistence of the left upper lobe tree-in-bud pulmonary nodules over a five month period A. Initial image of the right middle and lower lobe necrotic lung lesions; B. Improvement of the right middle and lower lobe lesions seen on repeat imaging five months later; C. Initial image of left upper lobe tree-in-bud pulmonary nodules; D. Redemonstration of grossly stable left upper lobe tree-in-bud pulmonary nodules seen on repeat imaging five months later

Of note, the patient had two recent admissions to our hospital prior to the abovementioned presentation, which will be briefly summarized to provide a more comprehensive clinical context. Six months prior to the patient's presentation with the left upper extremity skin lesions, the patient presented to our hospital with chest tightness and dyspnea secondary to decompensated heart failure and was found to be in cardiogenic shock requiring temporary mechanical support. During this first admission, his hospital course was complicated by the reactivation of herpes simplex virus-1 infection with mucocutaneous manifestations. He also had a computerized tomography (CT) scan of the chest, which revealed some tree-and-bud nodules in the left upper lobe and some necrotic lung lesions determined to be secondary to a recently treated hospital-acquired pneumonia (Figure 2A, 2C). Acid-fast cultures and fungal cultures from bronchoalveolar lavage and lung tissue returned negative. Lung tissue histology findings were not consistent with a fungal infection. An urgent pretransplant work-up included a positive T-SPOT®.TB test (Oxford Immunotec Global, Abingdon, United Kingdom), which was determined to be a false positive due to a lack of risk factors for tuberculosis infection and the possibility of *M. marinum *exposure as the reason for this positive test. Repeat T-SPOT®.TB test returned with an indeterminate result, followed by a negative result all during the same admission. The patient had no clinical signs or symptoms consistent with active *M. marinum* infection during this first admission. The patient was listed and eventually underwent a heart transplant during his first admission. After the transplant, the patient was started on an immunosuppressive regimen which included tacrolimus, mycophenolate mofetil, and prednisone which was tapered off over eight weeks. The patient was also started on a three-month course of valganciclovir prophylaxis, as both patient and his donor were cytomegalovirus (CMV) positive, as well as trimethoprim-sulfamethoxazole prophylaxis. The patient's first hospitalization lasted approximately two months, and then the patient was stable for discharge to home in good condition approximately ten days after undergoing OHT.

Three weeks post-OHT, and approximately ten days after being discharged from his first admission, the patient was readmitted to our hospital with left upper extremity edema, erythema, and tenderness thought to be acute bacterial cellulitis. During this second admission, he also was diagnosed with a large serous pericardial effusion requiring a pericardial window. It was presumed that the left upper extremity edema was likely a result of the large pericardial effusion. Blood cultures obtained during this second admission were negative, as were pericardial fluid bacterial, mycobacterial, and fungal cultures. The patient was empirically treated with piperacillin-tazobactam and linezolid with rapid symptom improvement. Piperacillin-tazobactam was discontinued after six days, and the patient completed a 10-day course of linezolid with significant symptom improvement; however, a small area of skin induration over the distal aspect of the medial forearm persisted despite treatment. The plan was to clinically monitor this indurated region with a plan for a possible skin biopsy if this persisted. The patient was again discharged home in good condition. 

Returning to this current presentation, given the patient's immunocompromised status, the patient was admitted to the hospital for a third time for additional work-up of his skin nodules. Two punch biopsies of the skin nodules were obtained. Given the patient's clinical stability, he was discharged shortly after the punch biopsies were obtained. Histopathology later revealed acutely inflamed granulation tissue with acid-fast stain positive for acid-fast bacilli (Figure 1B), consistent with a mycobacterial skin and soft tissue infection. Approximately 10 days after sample processing, tissue cultures grew organisms consistent with acid-fast bacilli, which later speciated to *M. marinum* (Figure 1C), with antimicrobial susceptibilities given in Table 1. 

**Table 1:** Antimicrobial sensitivities and minimum inhibitory concentrations for the patient's M. marinum isolate Note: A minimum inhibitory concentration was not performed for clofazimine.

Antibiotic	Sensitivity	Minimum Inhibitory Concentration (µg/mL)
Clofazimine	-	0.12
Ciprofloxacin	Resistant	>8
Moxifloxacin	Resistant	>4
Clarithromycin	Sensitive	2
Amikacin	Sensitive	4
Doxycycline	Intermediate	4
Minocycline	Intermediate	4
Trimethoprim-sulfamethoxazole	Resistant	>4/76
Linezolid	Sensitive	4
Rifampin	Resistant	2
Rifabutin	Sensitive	≤0.12
Ethambutol	Sensitive	≤0.125

Although without pulmonary symptoms, an outpatient CT scan of the chest revealed a slight interval decrease in the size of the right lower and middle lobe opacities and grossly stable left upper lobe tree-in-bud distribution pulmonary micronodules (Figure 2B, 2D). As his prior work-up did not reveal any infectious cause, we determined that the patient's diagnosis was consistent only with *M. marinum* skin and soft tissue infection, without definite evidence of disseminated disease. The patient was empirically started on treatment with clarithromycin 500 mg by mouth twice per day and ethambutol 1200 mg by mouth once per day while sensitivities were pending, and this treatment regimen was continued after sensitivities resulted. 

As seen in the clinical images (Figure 1D), the left upper extremity *M. marinum* infectious skin nodules responded readily to the treatment regimen. After beginning treatment, the patient returned to the infectious disease clinic for outpatient follow-up every two to three months for monitoring of his skin lesions and to assess for medication side effects. He returned for a total of four outpatient appointments to the infectious disease clinic. The patient was also very closely followed by his heart transplant team for post-transplant-related care. At the time of this writing, the patient's left arm nodules have fully resolved following six months of antimicrobial therapy. He tolerated the regimen well, with close monitoring and adjustment of tacrolimus dosing based on levels due to interactions with clarithromycin by his transplant team. Antibiotics were continued another month post-resolution of nodules, giving him a total of seven months of therapy. Near the end of his treatment course, we did consider a referral to the surgical service to assess for the removal of a deep skin nodule at the dorsal aspect of the left wrist, which was slow to respond. However, this eventually resolved as well, and neither imaging nor surgery was required. 

## Discussion

This case demonstrates an unusual clinical manifestation of *M. marinum* infection, given the delay in symptom presentation until several months after the patient's last known exposure. In a review of forty cases of *M. marinum* infection with known incubation times, the median and mean incubation periods were 21 and 30.1 days, respectively, and the 90th percentile for incubation periods was 60 days [11]. While incubation periods of up to nine months have been reported for *M. marinum* infections, cases with incubation periods over sixty days are quite rare, and there has been only one case with a documented incubation period of over ninety days [11]. The patient's last potential exposure occurred approximately five months prior to nodule development and two months prior to his episode of cellulitis, significantly longer than the average incubation period of *M. marinum* infection [11]. If the start of his infection is considered to be the development of skin nodules, there has only been one case of *M. marinum* infection described in the literature with a longer incubation period, the case of a 55-year-old man who developed cutaneous *M. marinum *infection approximately 270 days after exposure [11, 13].

*M. marinum* is a rare cause of infection in transplant recipients, with only a handful of cases documented in the literature [14, 15]. As in the immunocompetent population, the majority of cases in transplant recipients are linked to aquatic exposures [15]. Most patients developed limited cutaneous disease; however, disseminated infection has also been described [9, 15]. Cases have been documented in patients who have undergone kidney, lung, liver, pancreas, and stem cell transplants, but, after an extensive literature review, there are no documented reports in OHT recipients [9, 15]. Thus, this case appears to be the first case of *M. marinum* infection described in an OHT recipient.

There is not a well-established treatment regimen for *M. marinum* infections. In a French cohort study of 63 patients, all patients were treated with antibiotics, and 48% also received surgical intervention. The duration of antibiotic therapy ranged from one to twenty-five months, with a median treatment duration of three-and-a-half months [4]. Numerous different antibiotics, utilized as monotherapy or in combination, have been employed in the treatment of *M. marinum* infection, including rifampin, rifabutin, ethambutol, amikacin, tetracyclines, clarithromycin, trimethoprim-sulfamethoxazole, and fluoroquinolones [4, 16]. Commonly used drug combinations include clarithromycin and rifampin, clarithromycin and ethambutol, clarithromycin and a tetracycline, rifampin, and ethambutol, and rifampin and tetracycline [4, 17]. Rifampin, rifabutin, and clarithromycin have been found to have the greatest potency against *M. marinum* [18, 19]. Among newer agents, linezolid has also shown promise as a potential treatment option for *M. marinum* infection [19, 20]. Treatment failure of *M. marinum* infection is uncommon and has been estimated between 5.7% and 13% in cohort studies [4, 16-17]. Interestingly, treatment failure was not related to the antibiotic regimen used or the duration of antibiotic therapy, but it was associated with deeper infections [4]. The decision was made to more aggressively treat this patient with a two-drug regimen of clarithromycin and ethambutol, given his immunocompromised status as a transplant recipient.

This case also helps emphasize the importance of taking a thorough history, including a patient's current and prior hobbies and means of employment. This patient's positive T-SPOT®.TB test during his prior admission was attributed to a past history of *M. marinum* infection or exposure, given his occupation and recreational activities. However, in the absence of cutaneous lesions, active *M. marinum* infection was not considered at that time, and the patient did not receive any treatment for an *M. marinum* infection during that admission. Currently, there are no specific guidelines regarding the management of an asymptomatic patient with a positive T-SPOT®.TB test that is suspected to be due to *M. marinum* exposure.

Parikka et al. have demonstrated the reactivation of *M. marinum *infection in zebrafish exposed to γ-radiation, a model which aimed to mimic human immunosuppression [21]. Given the patient's considerable immunosuppressive medication regimen, prior occupational and recreational history, and last known epidemiological exposure two months prior to transplantation and five months prior to symptom onset, we hypothesize that these findings could suggest a similar reactivation phenomenon in a human host.

## Conclusions

This case represents an unusual presentation of an *M. marinum* skin and soft tissue infection in a heart transplant recipient with a fairly remote exposure history. Given these case findings and the reactivation phenomenon observed in irradiated zebrafish, it could be beneficial to have an increased suspicion of *M. marinum* infection in transplant recipients who develop new skin lesions in the post-transplant period. Additionally, it is important to have these patients closely follow up with a physician after a transplant to monitor for signs and symptoms of a reactivated *M. marinum* infection. This case sheds new light on the complexity and pathogenesis of *M. marinum* infection in the immunosuppressed population.
